# Vitreomacular interface abnormalities in patients with diabetic macular oedema and their implications on the response to anti-VEGF therapy

**DOI:** 10.1007/s00417-018-4009-6

**Published:** 2018-05-19

**Authors:** Michael Mikhail, Stephen Stewart, Felicia Seow, Ruth Hogg, Noemi Lois

**Affiliations:** 1 Department of Ophthalmology, Belfast Health and Social Care Trust, Belfast, UK; 2 Wellcome-Wolfson Institute for Experimental Medicine, Queen’s University, Belfast, UK

**Keywords:** Retina, Diabetic macular oedema, OCT imaging, Vitreomacular interface abnormalities, Intravitreal anti-VEGF injection

## Abstract

**Purpose:**

To determine whether the presence of vitreomacular interface abnormalities (VMIA) in patients with diabetic macular oedema (DMO) modifies the response to ranibizumab.

**Methods:**

Medical records and spectral-domain optical coherence tomography (SD-OCT) scans of consecutive patients with centre-involving DMO initiating therapy with ranibizumab between December 2013 and March 2014 at the Belfast Health and Social Care Trust were reviewed. Patients were identified through an electronic database. Demographics; systemic baseline characteristics; history of previous ocular surgery/laser; best-corrected visual acuity (BCVA), central retinal thickness (CRT) and stage of retinopathy at presentation; and BCVA, CRT and presence/absence of fluid at the last follow-up were recorded. OCT scans were reviewed by a masked investigator who graded them for the presence/absence of VMIA at baseline and during follow-up and for the change in the posterior hyaloid face during follow-up. The association between (1) VMIA at baseline and (2) the change in the posterior hyaloid face during the follow-up and functional/anatomical outcomes was evaluated.

**Results:**

One hundred forty-six eyes of 100 patients (mean age 63.5 years) followed for a mean of 9 months (range 2–14 months; only 9/146 dropped to follow-up before month 6) were included. Statistically significant differences were observed at baseline in BCVA (*p* = 0.007), previous macular laser and panretinal photocoagulation (PRP) (*p* = 0.006) and previous cataract surgery (*p* = 0.01) between eyes with and without VMIA, with better levels of vision, higher frequency of macular laser and lower frequency of PRP in eyes where no VMIA was present. Multivariable regression analysis did not disclose any statistically significant associations between VMIA at baseline or change in the posterior hyaloid face during the follow-up and functional and anatomical outcomes following treatment.

**Conclusion:**

VMIA are associated with worse presenting vision in patients with DMO; VMIA or change in the posterior hyaloid face during the follow-up did not modify the response to ranibizumab in this study.

## Introduction

The vitreomacular interface (VMI) gained greater scientific interest since the advent of optical coherence tomography (OCT). Spectral-domain OCT (SD-OCT) allows excellent visualisation of the VMI, enabling the study of the role of VMI abnormalities (VMIA) in the development of macular disease and the response to treatment. Vitreomacular traction (VMT), a form of VMIA [1], has been proposed as one of many aetiological factors for the development of diabetic macular oedema (DMO) [2]. Relief of traction, either spontaneously or through vitrectomy, may promote DMO resolution in some cases [3–5].

The reported prevalence of VMIA in patients with DMO ranges from 6.6 to 52.1%, depending on the diagnostic criteria and retinal imaging modality used [2, 6–10]. Only a few of these studies, however, used SD-OCT [9, 10]. Recently, Akbar Khan et al. used SD-OCT to image a cohort of patients with centre-involving DMO undergoing macular laser treatment and found a VMIA prevalence of 26% in this group [9].

Anti-vascular endothelial growth factor (anti-VEGF) agents have revolutionised the management of DMO. Clinical studies exploring the effect of the presence or absence of VMIA on the response to anti-VEGF treatment in DMO patients are scarce [10, 11]. Elucidating the effect of VMIA on the response to anti-VEGFs is important to guide the management of patients with DMO; if VMIA were associated with a worse response to treatment, early vitrectomy could be considered.

Intravitreal injections of anti-VEGFs (or other substances) may alter the anatomical structure of the vitreous leading to changes in the status of the posterior hyaloid [10, 12]. The posterior hyaloid, previously attached, could detach as a result of these changes, and this could potentially modify the course of DMO, even leading to its resolution.

Herein, we present findings on a large cohort of patients receiving intravitreal anti-VEGF therapy with ranibizumab for DMO and investigate the potential relationship between (1) VMIA at baseline and (2) the change in the posterior hyaloid face during the follow-up and functional/anatomical outcomes following anti-VEGF therapy.

## Methods

The medical records of consecutive patients with centre-involving DMO that met the eligibility criteria for intravitreal ranibizumab treatment as per the UK National Institute for Health and Clinical Excellence (NICE) guidelines [13] and who presented to the Macular Unit, Belfast Health and Social Care Trust, Belfast, UK, during the period between December 1, 2013, and March 31, 2014, were reviewed. Only patients with DMO naive to intravitreal injections of anti-VEGFs/steroids were included. Patients with a history of previous laser treatment were also included. Those with a history of pars plana vitrectomy were excluded.

In keeping with the standard clinical practice at our site, all patients initiating anti-VEGF therapy received an ocular examination, which included refraction undertaken by an optometrist and best-corrected visual acuity (BCVA) measured with Early Treatment Diabetic Retinopathy Study (ETDRS) visual acuity charts at baseline, visual acuity testing obtained using ETDRS visual acuity charts and, following the refraction obtained at baseline, intraocular pressure measurement and slit-lamp biomicroscopy. SD-OCT scans were routinely obtained by trained ophthalmic photographers at every clinic visit using commercially available equipment (Spectralis OCT; Heidelberg Engineering, Heidelberg, Germany). SD-OCTs were obtained in the standard mode (the enhanced depth imaging mode was not used for the purpose of evaluating DMO).

Grading of the diabetic retinopathy was determined by the ophthalmologists evaluating the patients in the clinic. Macular oedema was classified, based on OCT findings, into mild, moderate and severe as described by the Global Diabetic Retinopathy Project Group [14]. Grading of the DMO was determined by one of the investigators (MM) based on OCT and fundus images.

All intravitreal anti-VEGF injections were performed in a clean room with topical povidone 5% and topical anaesthetic before the procedure. The treatment protocol involved three-monthly ranibizumab injections followed by a pro re nata regimen thereafter.

### Evaluation of the vitreomacular interface

SD-OCT scans were used to obtain data on central retinal thickness (CRT) values. The CRT was computed automatically using built-in retinal mapping software. SD-OCT video clips (Spectralis OCT; Heidelberg Engineering, Heidelberg, Germany) from all included patients were evaluated by a senior investigator (NL) masked to clinical findings, including visual acuity and treatments received; the status of the posterior hyaloid and VMI at baseline and at the last follow-up was graded (see below) and recorded.

**Fig. 1:**
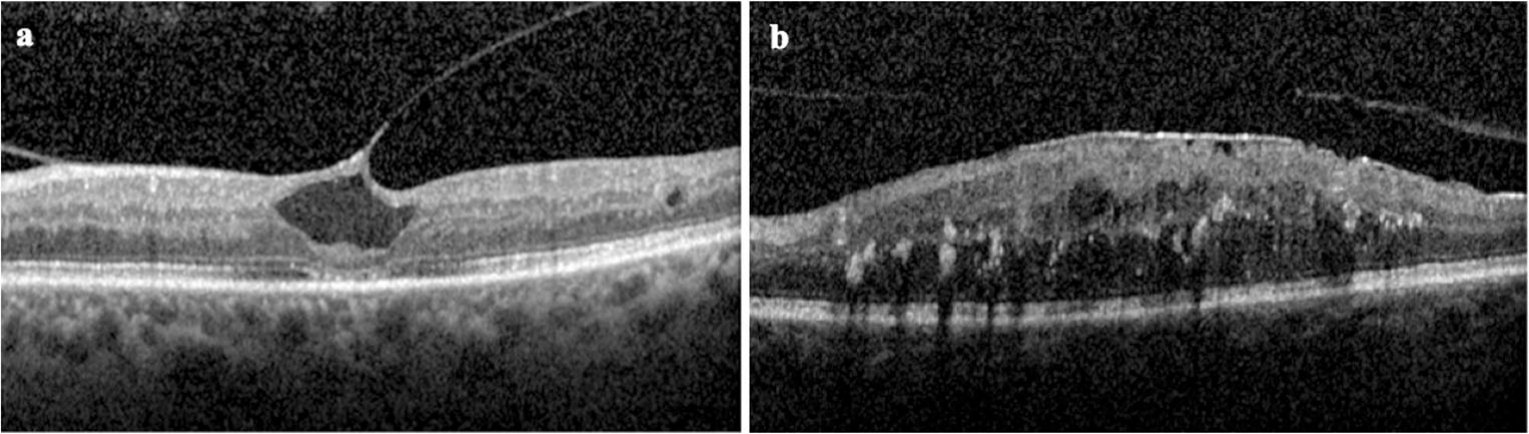
SD-OCT scan demonstrating VMIA, including VMT (**a**) and ERM (**b**) associated with DMO in two eyes of patients included in the study

SD-OCT video clips enabled detection of foveal and extra-foveal traction as well as changes outside the central macular area and also differentiation between epiretinal membranes and taut posterior hyaloid. The SD-OCT appearance of the inner retinal surface was graded based on the International Vitreomacular Traction Study Group classification of vitreomacular adhesion, traction, and macular hole as follows: (a) normal VMA, (b) VMT (Fig. 1a), (c) full-thickness macular hole (FTMH), (d) lamellar macula hole (LMH), (e) epiretinal membrane (Fig. 1b) and (f) combined epiretinal membrane (ERM) and VMT [1].

**Fig. 2:**
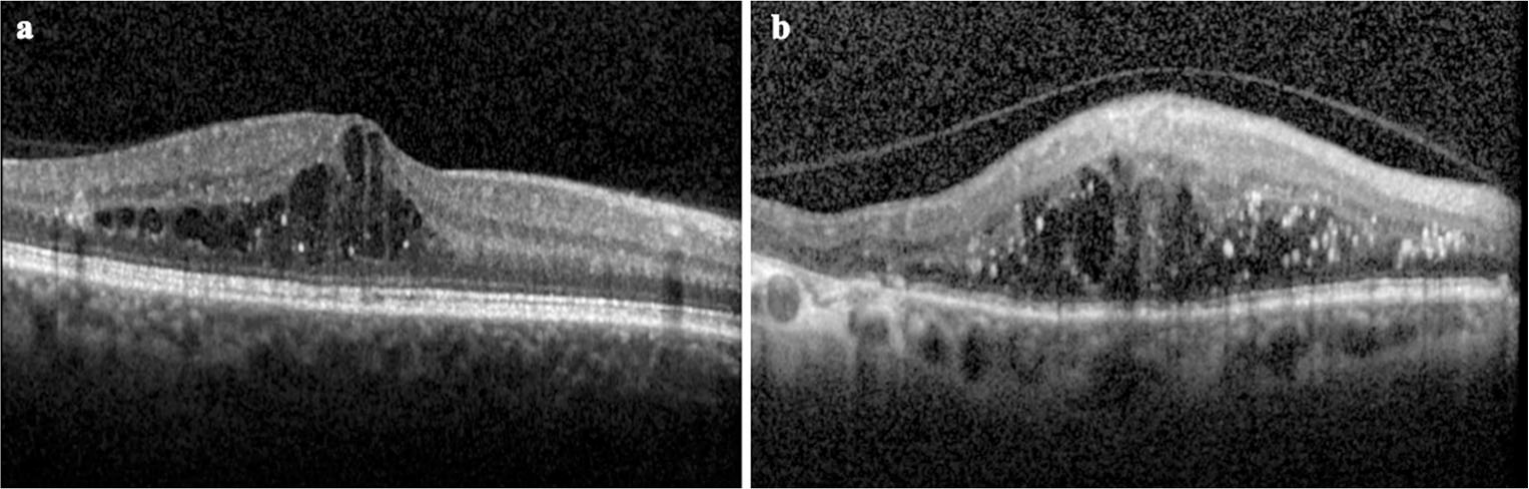
SD-OCT scans demonstrating two eyes included in the study with an attached posterior hyaloid (**a**) and with a detached posterior hyaloid (**b**)

The status of the posterior hyaloid face (PHF) was also graded based on the presence or absence of a PHF attachment as (a) totally attached (Fig. 2a), (b) partially attached, or (c) fully detached (Fig. 2b) at baseline. The occurrence of a partial or complete detachment of the PHF was also recorded at the last follow-up visit. The number of images with insufficient quality for grading as well as those in which determining the status of the PHF was not possible was also recorded.

### Statistical analysis

The statistical analysis of the data was performed using the Statistical Package for the Social Sciences, Windows version 21 (SPSS Inc., Armonk, NY). The distribution of continuous variables was assessed for normality using the Kolmogorov-Smirnov test, and the transformation was performed, if necessary, to achieve normal distribution. Categorical variables were compared using Pearson’s chi-square test. Continuous variables were compared using the independent samples *t* test.

General estimating equations (GEE) were used to undertake the multivariable regression analysis to enable data from both eyes to be included. GEE models account for the correlation between two eyes from one patient. A multivariable modelling controlled for age and gender was undertaken. A second model was also run controlling for those covariates shown to be significant in the univariate analysis. *p* values < 0.05 were considered statistically significant.

In patients with both eyes meeting eligibility criteria, data from both eyes were used. The authors confirm that data collection conformed to the local policy at the Belfast Health and Social Care Trust; this study was registered with the audit department (number 4966).

## Results

The study cohort included 146 eyes of 100 consecutive patients with a mean age of 63.5 years at the time of presentation (range 24–88 years). One hundred thirty-eight eyes (94.5%) exhibited severe macular oedema, which includes retinal thickening or hard exudates involving the centre of the fovea. Eighty-two eyes (56.1%) had no previous macular laser treatment at baseline. The descriptive characteristics of the 100 patients (146 eyes) studied are shown in Table 1; examples of the features investigated in the study are depicted in Figs. 1 and 2. Patients were followed up for a mean of 9 months (range 2–14 months). Eyes were treated with ranibizumab only (i.e. no other anti-VEGFs used) during this period. 9/146 eyes were lost to follow-up before month 6. The number of injections per month of follow-up was 0.8 (SD 0.3).

**Table 1:** Descriptive characteristics of the study cohort and univariable analysis comparing eyes with and without VMIA at baseline and eyes with and without a change in the posterior hyaloid interface during the follow-up

	All (*n* = 146 eyes)	Presence of VMIA (*n* = 28 eyes)	Absence of VMIA (*n* = 118 eyes)	p*^~^	Change in the posterior hyaloid interface during follow-up (*n* = 22 eyes)	No change in the posterior hyaloid interface during follow-up (*n* = 91 eyes)	p**^~^
Age (SD)	64 (13)	64 (14)	64 (13)	0.956	60 (15)	64 (13)	0.266
Male sex (%)	61	14 (50)	78 (66)	0.087	17 (74)	55 (60)	0.563
DM duration in years (SD)	17 (10)	18 (10)	17 (10)	0.540	16 (9)	17 (11)	0.729
HbA1C level in mmol/L (SD)	66 (16)	63 (17)	66 (16)	0.449	69 (18)	64 (15)	0.284
Baseline BCVA (SD)	53 (17)	45 (20)	55 (16)	*0.007*	58 (20)	52 (17)	0.170
Baseline central retinal thickness (SD)	536 (140)	522 (128)	539 (143)	0.580	533 (162)	541 (146)	0.814
% history of hypertension	90	90	91	0.528	87	90	0.726
% history of kidney disease	6	14	4	0.069	0	4	*0.019*
Stage of DR							
No DR (%)	0 (0)	0 (0)	0 (0)		0 (0)	0 (0)	
Mild NPDR (%)	43 (29)	5 (18)	38 (32)		7 (30)	24 (26)	
Moderate NPDR (%)	28 (19)	6 (21)	22 (19)		2 (9)	16 (18)	
Severe NPDR (%)	24 (16)	2 (7)	22 (19)	*0.035*	6 (27)	15 (17)	0.276
Very severe NPDR (%)	6 (4)	0 (0)	6 (5)		1 (5)	5 (6)	
Proliferative DR (%)	6 (4)	3 (11)	3 (3)		2 (9)	4 (4)	
Treated proliferative DR (%)	39 (27)	12 (43)	27 (23)		4 (18)	27 (30)	
Previous ocular surgery							
None (%)	116 (80)	17 (61)	99 (84)	*0.010*	17 (77)	74 (81)	0.783
Cataract surgery (%)	30 (20)	11 (39)	16 (16)		5 (22)	17 (19)	
Previous laser surgery							
None (%)	38 (26)	8 (29)	31 (26)		7 (30)	19 (21)	
Macular laser (%)	64 (44)	5 (18)	59 (50)		9 (41)	42 (46)	
PRP (%)	15 (10)	6 (21)	9 (8)	*0.006*	1 (5)	11 (12)	0.592
Macular laser + PRP (%)	28 (19)	9 (32)	19 (16)		5 (23)	18 (20)	
Posterior hyaloid status at baseline, *n* (%)							
Totally attached	20 (14)	0 (0)	20 (17)		6 (26)	14 (15)	
Partially attached	69 (47)	11 (39)	58 (49)		16 (70)	52 (57)	
Totally detached	22 (15)	8 (29)	14 (12)	*0.012*	0 (0)	22 (24)	< *0.001*
Could not be determined	30 (21)	9 (32)	21 (18)		0 (0)	1 (1)	
Ungradable	5 (3)	0 (0)	5 (4)		0 (0)	2 (2)	
VMIA							
Present, *n* (%)	28 (19)				19 (86)	75 (82)	0.210
Absent, *n* (%)	118 (81)				3 (14)	16 (18)	
Macular status at the final visit							
Dry, *n* (%)	23 (16)	4 (14)	19 (16)		6 (27)	11 (12)	
Fluid present, *n* (%)	118 (81)	24 (86)	94 (80)	0.746	15 (68)	77 (85)	0.182
Ungradable, *n* (%)	5 (3)	0 (0)	5 (4)		1 (5)	3 (3)	
No. of injections per month of follow-up (SD)	0.8 (0.3)	0.8 (0.3)	0.7 (0.3)	0.145	0.8 (0.3)	0.7 (0.3)	0.290
Change in posterior hyaloid during follow-up, *n* (%)							
None	91 (62)	16 (57)	74 (63)				
Complete or partial detachment	22 (15).	3 (11)	20 (17)				
Could not be determined*	29 (20)	9 (32)	20 (17)	0.210			
Ungradable follow-up images	4 (2)	0 (0)	4 (3)				

*p* < 0.05 is highlighted in italics. “Could not be determined”, not possible for the grader to determine whether the vitreous was fully attached or fully detached (reflectivity would not be clearly present or absent in the vitreoretinal interface of vitreous cavity). Ungradable, images of inadequate quality for evaluation

^~^Chi-square tests for categorical variables and independent *t* test for continuous variables

*p, comparison between patients with or without VMIA at baseline

**p, comparison between patients with or without a change in the posterior hyaloid face during the follow-up

### Factors associated with VMIA at baseline

Signs of VMIA were detected in 28 eyes (18.5%); 19 of these (70%) had an ERM, 8 (28.5%) had VMT and one (0.5%) had both VMT and ERM. A statistically significant difference in mean BCVA was observed between patients with and without VMIA, with patients with VMIA having worse vision (45 ETDRS letters in eyes with VMIA compared to 55 ETDRS letters in eyes without VMIA; *p* = 0.007). Eyes with VMIA were more likely to have undergone panretinal photocoagulation but less likely to have had macular laser than eyes without VMIA (*p* = 0.006). There was no association between CRT at presentation and the presence of VMIA. A higher proportion of patients with VMIA had undergone cataract surgery (*p* = 0.01). There was no association between the presence of VMIA and the duration of diabetes, HbA1C level, history of hypertension or kidney disease.

### Presence of VMIA at baseline and treatment outcomes

Table 2 shows the results of the multivariable regression analysis undertaken to evaluate associations between the presence of VMIA at baseline and treatment outcomes adjusted for age, gender, retinopathy stage at baseline and history of previous cataract surgery or laser therapy. No statistically significant associations between the presence of VMIA at baseline and outcomes following ranibizumab treatment, including the change in BCVA or CRT from the baseline to the last follow-up, and the presence or absence of fluid at the macula at the last follow-up were identified.

**Table 2:** Relationship between the presence of vitreous interface abnormalities at baseline and treatment outcomes during follow-up

Outcome	Model 1			Model 2		
	*β*	CI	*p*	*β*	CI	*p*
Change in BCVA	0.019	− 0.024–0.062	0.387	0.013	− 0.022–0.031	0.568
No. of injections per month of follow-up	1.100	− 0.021–0.048	0.222	1.978	− 1.457–0.878	0.175
Central retinal thickness	− 0.002	− 0.001–0.005	0.170	− 0.001	− 0.002–0.004	0.439
Macula status at the final visit	0.105	− 1.320–1.529	0.886	0.650	− 1.277–2.578	0.508

Model 1: corrected for age and sex. Model 2: corrected for age, sex, diabetic retinopathy stage at baseline, history of cataract surgery and history of previous laser therapy

### Development/resolution of VMIA during the follow-up

In this series, no eyes developed VMIA or had resolution of pre-existing VMIA during the follow-up.

### Factors associated with the change in the posterior hyaloid interface during follow-up

Partial or complete detachment of the posterior hyaloid interface was observed in 22 eyes during the follow-up (15%). A history of kidney disease (*p* = 0.019) was the only factor found to be statistically significantly associated with this occurrence.

### Treatment outcomes and changes in the posterior hyaloid surface during follow-up

Multivariable regression analysis did not reveal any statistically significant difference between detachment of the posterior hyaloid during follow-up and change in BCVA, number of injections per month, changes in CRT and status of macula at follow-up, when corrected for age, gender and history of kidney disease (Table 3). There was an association of borderline significance (*p* = 0.052) between posterior hyaloid detachment and absence of macular fluid at the last follow-up.

**Table 3:** Relationship between the presence of change in posterior hyaloid during follow-up and treatment outcomes

Outcome	Model 1			Model 2		
	*β*	CI	*p*	*β*	CI	*p*
Change in BCVA	0.009	− 0.036–0.155	0.694	0.009	− 0.036–0.053	0.707
No. of injections per month of follow-up	1.318	− 0.772–3.408	0.216	1.241	− 0.820–3.302	0.238
Central retinal thickness	0.000	− 0.003–0.016	0.901	0.001	− 0.003–0.034	0.854
Macula status at the final visit	− 1.109	− 2.325–0.107	0.074	− 1.247	− 2.506–0.013	0.052

Model 1: corrected for age and sex. Model 2: corrected for age, sex and history of kidney disease

## Discussion

In the current study, VMIA, as defined by the International Vitreomacular Traction Study Group, were detected in 18.5% of patients with DMO, the majority of these being ERM (70%). VMIA were more common in patients that had previously undergone panretinal photocoagulation (PRP) and cataract surgery and less frequent in those that had received macular laser. Patients with VMIA presented with lower levels of vision. The presence of VMIA at baseline did not dampen the functional (VA) or structural (CRT) response to ranibizumab treatment.

### VMIA in patients with DMO

The prevalence of VMIA in patients with DMO identified in this study appears to be within the previously reported range (Table 4) [2, 6–10]. It is lower than that reported in a recently published study, which found an ERM prevalence of 43% in a group of 77 patients (104 eyes) [10]. This study included eyes with higher stages of diabetic retinopathy (DR) at presentation [10].

**Table 4:** Prevalence of vitreoretinal interface abnormalities in DMO patients

Study	Study design	Sample number (patients/eyes)	Vitreoretinal anomaly
Kim et al. [6]	Retrospective, observational	119/164	OCT revealed morphological patterns of DMO patients with PHT (15.6%)
Ghazi et al. [2]	Prospective, observational case series	25/48	ERM or anomalous VMA in 52.1%
Ophir et al. [7]	Retrospective study	122/186	VFT in 25 eyes (13.4%) and extra-foveal traction in 20 (10.8%)
Chang et al. [8]	Retrospective, observational	76/96	16 eyes (6.6%) had ERM or anomalous VMA
Akbar Khan et al. [9]	Retrospective, observational	198/198	Partial vitreomacular separation in 12% and ERM in 14%
Wong et al. [10]	Prospective, observational	77/104	43% had foveal-involving ERM and 63% had eccentric ERM
Mikhail et al. (current study)	Retrospective, observational	100/146	VMIA in 28 eyes (18.5%): 19 with ERM, 8 with VMT and 1 with both VMT and ERM

*ERM* epiretinal membrane, *DMO* diabetic macular oedema, *PHT* posterior hyaloid traction, *TRD* tractional retinal detachment, *VMA* vitreomacular adhesion, *VFT* vitreofoveal traction

Previous PRP was associated with the presence of VMIA. ERM formation may occur as a result of laser panretinal photocoagulation [15]. It has also been proposed that PRP induces an angiofibrotic switch by lowering VEGF levels and increasing the ratio of connective tissue growth factor over VEGF, which may subsequently trigger changes in the vitreomacular interface [16]. However, as PRP is applied to patients with proliferative diabetic retinopathy, a causal relationship between the pathogenic events related to advanced stages of disease and the occurrence of VMIA cannot be ruled out. Interestingly, of those patients with VMIA, only 18% had previously had macular laser when compared with 50% of those without VMIA. It is not clear how macular laser could exert this apparent protective effect on the development of VMIA.

VMIA were also identified more frequently in patients who had undergone cataract surgery. Pseudophakia is associated with an increased incidence of posterior vitreous detachment [17]. Furthermore, epidemiological studies have established a link between ERM and previous cataract surgery [18, 19], and changes in the vitreomacular interface following cataract surgery in otherwise healthy eyes have been documented using OCT [20]. It is possible that in the context of DR and DMO, inflammation may promote a firmer attachment of the vitreous to the macular area. Under these circumstances, it is possible that, following cataract surgery, detachment of the vitreous outside the macula with incomplete separation at the macula would lead to VMIA.

Lower visual acuity at presentation was found in patients with VMIA when compared with those without them. This agrees with findings of other studies [6, 11].

### Functional and structural outcomes following anti-VEGF in the presence of VMIA

Previous studies evaluating the effect of VMIA on the response to anti-VEGF agents have reported conflicting results. Further analysis of a Diabetic Retinopathy Clinical Research Network study found the absence of “surface wrinkling retinopathy”, as determined by colour fundus photography, to be associated with a significantly better visual outcome after one year of treatment with ranibizumab [21]. However, patients were included in this study only if DMO was the primary cause of vision loss and, therefore, patients with more severe VMIA would have been excluded.

A small study by Yoon et al. evaluated the effect of VMIA (defined as epiretinal membrane and/or anomalous vitreomacular adhesions) on the response to anti-VEGF treatment in patients with DMO [22]. Only ten eyes with VMIA, and five without, were included. Eyes with VMIA showed less improvement in BCVA after 3 anti-VEGF injections.

Wong and colleagues recently reported that the presence of an ERM at presentation was predictive of a more limited functional (visual acuity) and anatomical (CRT) response in a study including 77 patients (104 eyes) with DMO [10]. The prevalence of ERM in this study was high compared with that observed in the current study (43 versus 13%), and patients had higher stages of severity of diabetic retinopathy (80% with pre-proliferative and proliferative disease versus 51% with severe/very severe non-proliferative diabetic retinopathy (NPDR) or proliferative DR). In this study, no patients were classified as having VMT.

A retrospective cohort study of 124 eyes enrolled in the READ-3 trial found that eyes with evidence of vitreomacular adhesion at baseline (*n* = 26) had a greater improvement in visual acuity than those without VMA (*n* = 98) at 6 months following treatment with ranibizumab [12]. In this study, however, patients with VMT were excluded from the analysis.

A small retrospective case series including 31 eyes studied the effect of intravitreal injection of bevacizumab on visual acuity, central macular thickness and total macular volume after 3 months [23]. VMIA were defined as the presence of epiretinal membrane or vitreomacular traction on OCT; eight patients had VMIA. Patients with a history of previous macular laser were excluded. No statistically significant difference was found in visual outcome or retinal thickness in patients with or without VMIA.

A retrospective study of 142 patients (201 eyes) undergoing anti-VEGF therapy found the mean incidence of VMIA to be 6.43% per year [11]. In this study, patients with VMIA at baseline were excluded. It was found that patients with poor baseline visual acuity had a higher chance of developing VMIA during the follow-up. In accordance with our findings, there was no statistically significant difference with regard to visual acuity improvement and reduction in the macular oedema between eyes that developed VMIA and those that did not. VMIA were classified in the same manner as our study, although images were obtained only with time-domain OCT [11].

PVD is more prevalent in eyes without DMO [24]. There is also some evidence suggesting the potential beneficial effect of pars plana vitrectomy in promoting macular oedema resolution [3–5]. In our study, we found an association of borderline significance between detachment of the posterior hyaloid on follow-up and resolution of macular oedema.

This study has several limitations, including its retrospective nature, the relatively short follow-up period and the fact that cataract surgery during the study period was not systematically recorded. We believe, however, that it would be unlikely that patients included in this series would have received cataract surgery during the study period. The reasons for this are that it is a standard practice at our institution for patients with DMO to be treated first with anti-VEGFs and cataract surgery postponed until resolution of the DMO has occurred. Also it would be dffocult that paetients would hacve undergone cataract surgery suring the study period considering waiting times for cataract surgery and the length of the follow-up of the patients included in the study. Information about whether eyes received focal laser treatment or PRP during the period of the study was, similarly, not systematically recorded. It would be unlikely that patients would have received focal laser treatment during the study period as in our clinics, patients initiating anti-VEGF therapy would have continued on anti-VEGFs monthly, unless the fluid would have resolved, during the first year of treatment. A small proportion of eyes, however, would have received PRP for active PDR (*n* = 6; Table 1). Furthermore, although in this study VMIA did not appear to affect the response to anti-VEGF therapy, it remains unclear whether resolving the VMIA surgically in some cases could have yielded to even better functional results. A randomised clinical trial comparing anti-VEGF with anti-VEGF plus vitrectomy for the treatment of DMO associated with VMIA would be required to fully address this. The strengths of this study include the use of an electronic database to identify all patients examined and treated in our institution during the period of the study, the relatively high proportion of patients (eyes) included, the masked evaluation of OCT and the fact that the data presented was obtained from a clinical setting and, thus, it is likely that the results observed would be generalisable.

The findings of our study would suggest that the presence of VMIA in patients with DMO does not appear to dampen the response to anti-VEGF therapy.
